# The Importance of First Impressions: Early Events in *Mycobacterium tuberculosis* Infection Influence Outcome

**DOI:** 10.1128/mBio.00342-16

**Published:** 2016-04-05

**Authors:** Anthony M. Cadena, JoAnne L. Flynn, Sarah M. Fortune

**Affiliations:** a Department of Microbiology and Molecular Genetics, University of Pittsburgh School of Medicine, Pittsburgh, Pennsylvania, USA; b Immunology Graduate program, University of Pittsburgh School of Medicine, Pittsburgh, Pennsylvania, USA; c Department of Immunology and Infectious Disease, Harvard T. H. Chan School of Public Health, Boston, Massachusetts, USA; d Ragon Institute of MGH, MIT, and Harvard, Cambridge, Massachusetts, USA

## Abstract

Tuberculosis remains a major health threat in much of the world. New vaccines against *Mycobacterium tuberculosis* are essential for preventing infection, disease, and transmission. However, the host immune responses that need to be induced by an effective vaccine remain unclear. Increasingly, it has become clear that early events in infection are of major importance in the eventual outcome of the infection. Studying such events in humans is challenging, as they occur within the lung and thoracic lymph nodes, and any clinical signs of early infection are relatively nonspecific. Nonetheless, clinical studies and animal models of tuberculosis have provided new insights into the local events that occur in the first few weeks of tuberculosis. Development of an effective vaccine requires a clear understanding of the successful (and detrimental) early host responses against *M. tuberculosis*, with the goal to improve upon natural immune responses and prevent infection or disease.

## INTRODUCTION

Tuberculosis (TB) is the number one infectious disease killer in the world, responsible for 1.5 million deaths in 2014 (1). There were an estimated 9.6 million new cases of active (contagious) TB in 2014 worldwide (1). The numbers of active cases are only the tip of the iceberg, as the majority of humans infected with *Mycobacterium tuberculosis* control the infection and do not progress to active TB. Most of these people remain infected although asymptomatic; this is termed latent TB infection (or LTBI), and it is estimated that more than 2 billion humans have latent TB. People with latent TB are at risk for development of reactivation TB at some later time point, and they are therefore potential reservoirs for transmission. In recent years, it has become appreciated that *M. tuberculosis* infection presents across a clinical spectrum (2). Active TB can present as mild, moderate, or severe disease, with a variety of pathological features. Similarly, clinically latent infection appears to also be a spectrum of outcomes, from presumably cleared infection to subclinical disease without overt symptoms (3). The risk of reactivation is estimated at 10% at a population level. However, it is likely that some humans with LTBI have essentially no risk of reactivation (e.g., persons with cleared or very-well-contained infection), whereas the risk is much higher for those individuals with subclinical or percolating disease.

*M. tuberculosis* infection generally progresses quite slowly, with active TB being diagnosed most commonly within the first 2 years of infection. People often do not know when they are exposed or infected and, therefore, studying early events of infection in humans can be challenging. Nonetheless, some studies support that the earliest events in *M. tuberculosis* infection are critically important in dictating clinical outcome. First and foremost, the inoculum dose in animal models from mice to nonhuman primates influences the severity of infection, so that more bacteria delivered to the lungs results in a worse outcome. In addition, data from nonhuman primate studies suggest that between 3 and 6 weeks postinfection, one can predict whether the animal will progress to active TB disease or remain latently infected 6 to 9 months later, based on the extent of early dissemination and serologic (erythrocyte sedimentation rate [ESR]) and positron emission tomography-computer tomography (PET-CT) features suggestive of more extensive inflammation (4). Thus, the clinical course is at least in part dictated by the early innate and adaptive immune responses.

Indeed, efforts to understand the basis of protective immunity to *M. tuberculosis* infection are pushing us to look even earlier into the course of infection. Clinical studies suggest that there are individuals who are highly exposed to *M. tuberculosis* but remain persistently tuberculin skin test (TST) negative, and thus presumably uninfected. These individuals raise the possibility that at still-earlier points in the course of infection, it is possible for the host to fully clear the bacteria and that this early bactericidal response could be harnessed through vaccination.

In view of the emerging importance of these initial events for clinically relevant outcomes, we here review the literature on early infection in tuberculosis.

## CLINICAL DATA ON EARLY INFECTION

There are major gaps in our understanding of the early events in *M. tuberculosis* infection in humans. These stem from the difficulty in identifying the onset of infection, for which there are no good diagnostic tools. The classic diagnostic test for *M. tuberculosis* infection is the TST, which is a delayed-type hypersensitivity response to a crude mixture of *M. tuberculosis* proteins and lipids known as purified protein derivative (PPD). A more recent test for infection is the interferon gamma (IFN-γ) release assay (IGRA), in which blood is stimulated with two *M. tuberculosis*-specific antigens and then assayed for IFN-γ by enzyme-linked immunosorbent assay or enzyme-linked immunosorbent spot assay. Both the TST and IGRA measure the T cell response to mycobacterial antigens and therefore are not positive until a measurable T cell response has been induced. Induction of measurable T cell responses to *M. tuberculosis* infection can be quite slow. It occurs in humans approximately 6 weeks postinfection (5). In nonhuman primates and in guinea pigs, TST conversion occurs 4 to 8 weeks after exposure and infection (6, 7). Although the TST is not usually performed in mice, an older study showed that PPD^+^ skin test responses were observed at 4 to 6 weeks postinfection (8). However, *M. tuberculosis*-specific T cell responses in lymphoid tissues can be measured as early as 2 weeks postinfection (9). Conversion of a negative to positive TST or IGRA result denotes recent exposure and infection. Frequent TST or IGRA testing is necessary in clinical settings to establish an approximate time of infection, but by the time a T cell response is measurable, the early events in infection have already occurred. The lack of simple diagnostic tests that can be used to identify infected individuals immediately after infection makes it difficult to conduct rigorous studies on the course of early infection in humans.

Despite these limitations, landmark studies were conducted by Poulsen in the Faroe Islands in the pretreatment era of the 1930s and 1940s that provided critical insights into initial infection in humans (5, 10). In these studies, a version of tuberculin skin testing was performed on nearly all of the 30,000 residents of the numerous villages in the Faroe Islands, and nearly all persons with active TB were known. Thus, the local epidemiology of TB in each village was well documented, and since most villages were small, tracking individuals and obtaining detailed histories were possible. New TST conversions were followed closely, with particular attention paid to identifying the time of infection by determining the index case and the duration of exposure to that case by the newly infected individual, as well as clinical signs postinfection, including X-ray and fluoroscopy findings. The first detailed description of these studies is a fascinating series of “case reports” documenting the duration of and time since exposure to an index case, skin test conversion, subsequent clinical manifestations, and outcome (5). In some cases, exposure to a person with active TB for less than 24 h resulted in TST conversion and subsequent development of primary TB. TST conversion was generally evident by 6 weeks postexposure. Interestingly, nearly all of the reported cases experienced a fever (termed “initial fever”) around the time of skin test conversion, and follow-up indicated hilar adenopathy, with a number of subsequent cases of active TB and deaths.

In a follow-up study, Poulson rigorously analyzed early symptoms of infection and outcome (10). A total of 232 subjects (children and adults) who had TST conversions within the 6-month interval of testing were followed for several years. An additional 285 subjects who were selected as presenting with an initial fever in conjunction with recent skin test conversion were also followed. Of the 232 “unselected” subjects, 63% had an initial fever after infection, the frequency of which was similar between adults and children. The fevers were of varied intensities and durations, though these differences were not predictive of disease outcome. These findings suggest an initial inflammatory process in the majority of those infected with *M. tuberculosis*, regardless of infection trajectory (i.e., primary disease or containment). Poulson documented other signs of initial inflammation associated with infection, including elevated ESR, a nonspecific sign of inflammation, and erythema nodosum, an inflammatory process often linked to mycobacterial infection. An elevated ESR was coincident with initial fever, and in most cases the ESR returned to normal within 2 months. Even with the X-ray and fluoroscopy technology available at the time, hilar adenopathy, presumably signifying thoracic lymph node (LN) enlargement, was identified in 55% of subjects unselected for fever in the first 2 months postinfection, with little increase in hilar lymph node involvement after this time. Pulmonary infiltrates were observed within the first year in 27% of converters, which agrees with findings of a separate study in Norway (11). These infiltrates were usually seen within 3 months of TST conversion and usually unilateral. Most of these infiltrates regressed over the next several months, and only 15% of those with infiltrates progressed to active TB. However, when data were separated between adults and children, the authors found that 2% of children with infiltrates developed active disease, whereas 25% of adult converters with infiltrates progressed to TB, suggesting that early pulmonary infiltrates in adult converters is linked to disease progression (10).

Importantly, the earliest stages of infection may also be associated with bacterial carriage in sputum. For example, a recent study employing active case finding to estimate the prevalence of TB in household contacts revealed a stunningly high rate of asymptomatic carriage of *M. tuberculosis* bacteria in the sputum in household contacts, who were presumably more recently infected by the incident case (12). Similarly, pediatric studies have suggested that there can be an early period of bacterial “excretion” after infection, which subsequently can resolve and does not necessarily herald eventual tuberculosis disease (13). This supports a model in which an early period of bacterial growth is relatively common, even where the infection will subsequently be controlled.

These studies provide evidence of an early evolution of the infection in the majority of those infected, including an inflammatory process, evidence of thoracic lymph node involvement, and potentially also the presence of culturable bacilli in the airways or sputum. Thus, the initial events in humans are not usually “silent” and suggest that the host immune response to initial infection is relatively robust. As most infections do not progress to active TB, this immune response is often successful in restraining the infection, although it is apparently a matter of months before this containment is complete.

These findings from human studies were recapitulated in our studies in macaque models of tuberculosis. Cynomolgus macaques develop active TB or latent infection, defined clinically, following infection with <25 CFU of *M. tuberculosis* strain Erdman (7, 14). Using PET-CT imaging with fluorodeoxyglucose (FDG) as a probe, immunological assays, and clinical assessments, we have demonstrated that all macaques infected with a low dose of *M. tuberculosis* have an evolution of infection within the lungs, with granulomas visible by 2 to 3 weeks based on PET-CT imaging (4, 15). Thoracic lymph nodes are often enlarged or show FDG avidity within a few weeks of infection. Using cultures of gastric aspirates and bronchoalveolar lavage (BAL) fluid samples as surrogates for sputum cultures, 30% of infected monkeys were found to shed culturable *M. tuberculosis* within 2 months of infection, indicating bacilli in airways during acute infection (14). This was loosely correlated with outcome, with 90% of macaques that progress to active TB showing a positive gastric aspirate or BAL fluid sample in the first 2 months of infection, compared to 44% of those monkeys that would present with latent infection (unpublished data). ESR is very low in uninfected monkeys (normal, <2 mm). An increased ESR within 60 days of infection is strongly correlated with eventual progression to active TB; an ESR of >15 in the first 2 months of infection correctly predicts outcome with 92% accuracy (unpublished data). Coleman et al. showed that formation of new granulomas between 3 and 6 weeks postinfection, and increased PET avidity in those granulomas, was associated with eventual development of active TB (4). Although radiographs are less useful in macaques than in humans and are not as sensitive as PET-CT, our early studies using X-rays suggested that early pulmonary infiltrates were observed in 60% of our macaques (7), most of which went on to develop active disease. The macaque data support the human data on early inflammatory responses following infection, but those data also suggest that the final outcome of primary infection is determined early by the ability of the host to control infection in the granulomas and prevent early dissemination.

A more-in-depth study of granulomas from macaques demonstrated that the bacterial burden in the initial granulomas at 4 weeks postinfection is relatively high (~5 × 10^4^ CFU) with minimal bacterial killing occurring (16). However, as the adaptive immune response is induced, bacterial killing in granulomas increases, dramatically reducing bacterial burden and in some cases sterilizing the granuloma. Indeed, even in monkeys that progress to active TB, an average of 30% of the granulomas are sterile (16). The specific factors that drive an individual granuloma toward a particular fate remain poorly understood, but they are likely to reflect the contributions of both host and bacterial factors.

### Early granuloma formation and bacterial dissemination.

What is known about the course of granuloma formation in people? Our understanding of early granuloma formation is rooted in results from human autopsy series published by Georges Canetti in 1955 (17). Based on his histopathologic examinations of 1,500 autopsies specimens, Canetti proposed that all tuberculous lesions proceed initially through an exudative and then a caseous phase. He suggested that lesions then sit at a “crossroads,” with some remaining solid and ultimately sclerosing and others softening, where softening of the caseum, not caseation *per se*, was the harbinger of active disease (17). At the time that Canetti published these descriptions, he did not understand the basis of the softening, although in about half the cases, it was associated with an influx of neutrophils. In a discussion that remains highly relevant today, he wrote that “this poses the question of whether an unknown factor orients the lesion from the outset toward liquefaction; that is, determines its fate from the earliest phases when the lesion may not be distinguished from another whose evolution will be ‘normal.’ On the other hand, one also often observes softening of lesions of long-standing radiologic existence; this is evidence of the potential danger of old caseous lesions.”

**FIG 1:**
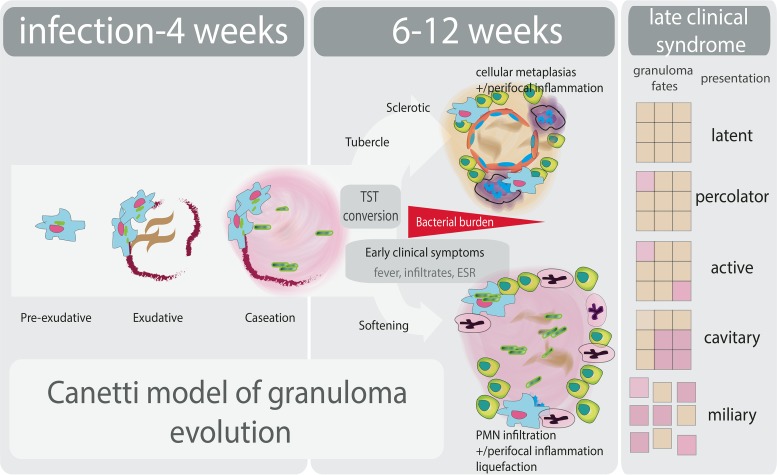
Granuloma formation (informed by Canetti’s seminal studies [17]) and progression coupled with outcome of *M. tuberculosis* infection.

Researchers have sought to better define and identify determinants of granuloma fate in a variety of experimental models. Data from these models have supported many of Canetti’s observations (Fig. 1). Exploration of early granuloma organization and development in guinea pigs has shown that within 21 days there is evidence of caseation preceding initiation of T cell responses, implicating both innate mechanisms and bacterial processes in early cellular death (18). Similarly, in macaques, the earliest granulomas isolated from the lungs (at 3 and 4 weeks postinfection) are primarily caseous in nature (19), where some of these lesions are expected to progress but most will not and, indeed, some will be fully sterilized after the onset of adaptive immunity.

The first steps in the development of the granuloma and the path to early caseation have been elucidated using *Mycobacterium marinum* infection in a zebrafish model. These studies indicate that early macrophage death in the primary granuloma is linked to bacterial replication, dissemination, and secondary granuloma formation (20, 21). Specific modulation of macrophage apoptosis and necrosis by the ecosanoid pathway compounds prostaglandin E2 (PGE2) and lipoxin A4 (LXA4), respectively, early in infection have been shown to influence infection outcome (22, 23). These studies extend the concept of the granuloma as a protective host response to suggest that it also provides a niche for mycobacteria to establish robust infection.

The events that dictate the fate of the granuloma after early caseation are less well understood. *M. tuberculosis* expresses matrix metalloproteinases that can degrade collagen and facilitate tissue destruction (24–26). The relative contribution of these bacterial virulence factors to the softening of the caseum that Canetti described, as opposed to caseation *per se*, remains unclear. It is possible that this reflects the effect of the enzymes responsible for the lipid mediators already implicated in TB disease, where alteration of activity is also expected to impact the linked lipid mediators of resolution of inflammation and neutrophil recruitment.

## WHAT ARE THE CRITICAL CELLULAR EVENTS IN EARLY INFECTION?

### Encounters with phagocytic cells in early infection.

Our current understanding of the cellular events in early infection of *M. tuberculosis* draws on a number of *in vitro* model systems and *in vivo* small animal model studies. Inhalation of a small number of *M. tuberculosis* organisms via droplet nuclei from a person with active tuberculosis results in deposition of the bacilli into the alveolar space and initial contact and phagocytosis by alveolar macrophages (27). Following infection, an early influx of phagocytic cells, including alveolar macrophages, neutrophils, and dendritic cells (DCs), arrive to this focus and begin to seed formation of a granuloma (28, 29). Studies using an *M. marinum* zebrafish model helped to characterize the earliest cells recruited to the site of infection and showed that macrophages, not neutrophils, were the dominant phagocytosing cells in the first 4 days postinfection (30). Murine studies with both *M. tuberculosis* (31) and *Mycobacterium bovis* BCG (32) suggested that there is early infection of alveolar macrophages, various populations of DCs, and neutrophils (33). At 2 weeks postinfection, the lungs of C57BL/6 mice infected with ~100 CFU of *M. tuberculosis* Erdman revealed that the predominant populations in the lungs were Ly6G^+^ neutrophils and F4/80^+^ macrophages (33). Further characterization of the kinetics of lung recruitment within the first 2 to 4 weeks of infection in mice infected with green fluorescent protein (GFP)-labeled *M. tuberculosis* revealed a diverse and dynamic interplay between host cells and bacteria (31). At 14 days postinfection, myeloid DCs, alveolar macrophages, and neutrophils had the highest percentage of *M. tuberculosis*-infected cells (GFP^+^); by day 21, neutrophils and myeloid DCs contained the highest percentages of bacilli. Similar observations of the mediastinal LNs determined that myeloid DC migration was the primary infected source during the initial stages of infections (31). These observations highlight the diverse range of early phagocytic responders to *M. tuberculosis* infection and implicate their initial influence on the progression of early disease and dissemination.

A recent review by Srivastava et al. comprehensively assessed the diversity of these initial mononuclear cell subsets and detailed their contributions in tuberculosis (34). Those authors evaluated the relative differences of each subset’s ability to both prime adaptive responses and control *M. tuberculosis* infection by reviewing each subset individually, drawing on *in vitro*, mouse, and human data*.* Ultimately, the authors posited that the inherent functional differences of each subset likely contribute to the overall outcome of infection following potential skewing of one subset over the others. Importantly, this hypothesis helps to bridge the observed diversity in cell types with the resulting clinical variability observed in tuberculosis. Building on this hypothesis, we propose that the interplay of these diverse cell subsets with *M. tuberculosis* in the initial stages of infection contributes to granuloma fate and heterogeneity (16, 35), which subsequently influences host outcome (4). From this, we hypothesize that the local differences driven by early host-pathogen interactions manifest as a spectrum of granulomas with varied capacities for containment and bacterial killing, which ultimately lead to different clinical outcomes. As a point of speculation, it is possible that the activation status of the initial infected cell that seeds a granuloma could set the stage for inflammation, T cell recruitment, macrophage activation, and the eventual fate of that granuloma.

### Neutrophil activity is a strong correlate of the outcome of human infection with *M. tuberculosis.*

In recent years, it has become clear that neutrophil activity is a strong correlate of human TB disease state. Studies of gene expression in the blood of people with active and latent TB revealed a signature of neutrophil-driven beta interferon (IFN-β) production in those with active disease (36, 37). Furthermore, neutrophils were the primary cells infected with *M. tuberculosis* in samples of human BAL fluid, sputum, and pulmonary cavities from persons with active TB cases (38). In addition to being a correlate of disease state, recent studies in small animal models have suggested that neutrophil activity directly contributes to the progression to active disease. For example, Dorhoi et al. sought to understand the role of microRNA223, which was identified as a correlate of *M. tuberculosis* infection state in a large human cohort (39). By studying the function of this microRNA in mice, they found that it controlled neutrophil recruitment to the lung during infection by regulating the expression of key neutrophil chemoattractants, including CXCL2 and CCL3, as well as interleukin-6 (IL-6). In spite of these observations, the role of neutrophils in early infection remains incompletely understood. In zebrafish, neutrophils contributed an early protective effect against *M. marinum* through NADPH-dependent oxidative killing (40). In mice infected with *M. tuberculosis*, early depletion of neutrophils reduced migration of DCs to lymph nodes and further delayed priming of antigen-specific CD4^+^ T cells (41). In the same study, DCs that ingested infected neutrophils migrated better in an *in vitro* transwell chemotaxis system than those that simply ingested free *M. tuberculosis* bacilli. These findings suggest that neutrophil recruitment plays a causal role in disease progression, with potentially both protective and destructive properties, that is likely dependent on their timing and magnitude of response.

Recent data suggest that the human gene expression signature of active tuberculosis, neutrophil-derived IFN-β expression, reflects part of a broader signaling network that regulates the function of key immune players, including macrophages, T cells, and neutrophils (42–44). The signaling network includes cytokines, most notably type I IFNs, IL-10, IL-1, and IL-1 receptor antagonist (IL-1RA), as well as lipid-derived small-molecule regulators of inflammation, such as the eicosanoid PGE2. Recent work by Mayer-Barber and colleagues suggested a cross-regulatory network in which type I IFNs promote the production of IL-10 and IL-1RA, which in turn negatively regulate IL-1 expression and IL-1-dependent expression of PGE2 (45). In this model, PGE2 and IL-1 are protective and inhibit bacterial growth. Alteration of this signaling network, for example by supplementing with PGE2 or increasing PGE2 by inhibiting 5-lipoxygenase (5-LO) with the asthma drug zileuton, dramatically ameliorates the infection outcome in mice. The mechanism(s) by which this signaling network controls the outcome of TB infection in infected animals or humans is unclear. These eicosanoids are important players in a cascade of lipid mediators that coordinate inflammation and the resolution of inflammation and include the prostacyclins, leukotrienes, thromboxanes, and resolvins (46). Importantly, these lipid mediators are both synthesized by—and have substantial effects on—other key immune cells in the granuloma, including neutrophils. The 5-LO product, LTB4, which has been implicated in zebrafish as an important driver of poor TB outcome (47, 48), is a powerful neutrophil chemoattractant that serves to amplify primary danger signals and coordinate neutrophil recruitment. Thus, it is likely that the profound effects of perturbing the type I IFN–IL-1–PGE2 signaling network in mice reflect not only the impact on macrophage fate but also the arrival and function of neutrophils at the site of infection.

### T cells and beyond.

For a granuloma to function, T cells are required. The interactions between *M. tuberculosis* and the cells of the innate immune system clearly have profound consequences on the subsequent adaptive immune response. The adaptive response is slow to emerge in *M. tuberculosis*-infected hosts. Humans (and macaques) convert a tuberculin skin test, a measure of an adaptive T cell response, at approximately 6 weeks postinfection (5, 7). T cell responses in blood can be detected in macaques between 4 and 6 weeks (14), while a T cell response in the lymphoid tissues of mice can be detected between 14 and 21 days postinfection (9, 49). This delay in T cell response has been attributed to several factors, including a delay in delivery of bacteria or antigens to the thoracic lymph nodes for T cell priming. Although some studies have suggested that the delay is in part due to the low numbers of bacilli delivered to the host (9), another study found that dose of infection did not appreciably influence the time to priming of an adaptive response (50). There may be specific bacterial factors that inhibit delivery of *M. tuberculosis* to the lymph nodes. Evidence exists for both dendritic cells and CCR2^+^ macrophages as important players in transit of *M. tuberculosis* to the lymph nodes for priming T cells (51, 52). Whether the bacteria that end up in the lymph nodes come from the airways during early infection, or the lung granulomas once they are established, is not yet clear. There is evidence from mice that dendritic cells can carry *M. tuberculosis* from airways to the thoracic lymph nodes (51). It is less clear where the CCR2^+^ macrophages encounter the bacilli for transport to lymph nodes, but it appears more likely that these are lung parenchymal bacteria. Nonetheless, most mouse studies have shown that *M. tuberculosis* bacilli must be in a lymph node to initiate priming of a T cell response (9, 49), although mice devoid of lymph nodes and spleens were capable of priming T cell responses in the lungs (53).

Alteration of macrophage apoptosis, driven by the balance of LXA4 and PGE2 signals, alters CD8^+^ T cell cross-priming by DCs (23). These seminal findings provide insight into earlier observations of *M. tuberculosis*-specific impairment of antigen presentation and defects in Ag85-specific CD4^+^ T cell expansion in spite of enhanced airway LPS-stimulated macrophage recruitment to the lung and increased migration of DCs to the draining LNs (9). In addition to delaying potent T cell responses, recent work in mice has proposed that preliminary *M. tuberculosis* dissemination utilizes CD11c^+^ DCs to seed new granuloma formation (54). Investigating both intraperitoneal BCG and aerosol H37Rv infections in C57BL/6 mice, Harding et al. demonstrated that inflammatory DCs are a possible source of bacterial spread after acute infection, as these cells are frequently arrested during their migration to the lymph nodes following interaction with antigen-specific T cells (54). These areas of infected DC-T cell capture generate new foci of inflammation that can either formulate new granulomas or extend preexisting structures depending on the distance traveled. Collectively, these findings support the view that virulent *M. tuberculosis* actively subverts the early host immune response by modulating preliminary macrophage death to delay the onset of potent adaptive responses and utilize trafficking of DCs to further dissemination. These adaptations are likely crucial for *M. tuberculosis* to establish a foothold for infection, given its slow growth.

In considering potential important drivers of granuloma resolution—or the softening of the caseum observed by Canetti—it is also interesting to note that the T cell response directly regulates the innate inflammatory response. Nandi and Behar showed in a mouse model that IFN-γ produced by CD4^+^ T cells inhibited neutrophil recruitment such that influx of neutrophils was reflective of a failed Th1 response (44). These results were extended by Mishra and colleagues, who showed that IFN-γ-dependent nitric oxide production suppressed IL-1 production by inhibiting assembly of the NLRP3 inflammasome (55).

In addition to phagocytes, *M. tuberculosis* likely encounters other cell types, cytokines, and innate defense molecules in the airways during initial infection. Mucosal-associated invariant T cells (MAITs) are CD3^+^ CD8^+^ (or double-negative) T cells that have T cell receptors encoded by the TRAV1/1 genes and are restricted not by the classical major histocompatibility complex molecules, but by a nonclassical molecule MR-1 (reviewed in reference 56). These cells are found at higher frequencies in blood and mucosal sites in humans than in mice. MAITs emerge from the thymus with effector capabilities and thus can be considered early responders to bacterial, including *M. tuberculosis*, infections. MAITs respond to cells infected with bacterial pathogens without prior exposure to that pathogen, produce the cytokines IFN-γ and tumor necrosis factor (TNF), and are cytotoxic. Although the range of microbial ligands recognized by these “innate” T cells is not known, it was shown that MAITs recognize microbe-derived riboflavin metabolites (57). Recent studies suggest greater T cell receptor (TCR) diversity than originally appreciated, and thus MAITs are likely to recognize other microbe-derived ligands (58). In MR-1-deficient murine models, MAIT cells were associated with early protection against bacterial pathogens, including mycobacteria (59). Thus, these cells may act as early sensors of *M. tuberculosis* infection in airways and provide early cytokines to activate macrophages against this infection.

Other innate cells, including natural killer (NK) cells, may also play a role in early *M. tuberculosis* infection. NK cells are strong producers of IFN-γ and TNF and can also be cytolytic for *M. tuberculosis*-infected macrophages (60). Mycolic acids are ligands for NK cells, and human studies have shown substantial variability of responses by NK cells to extracellular *M. tuberculosis* (61), suggesting that the capacity of NK cells to recognize and respond to *M. tuberculosis* could contribute to early innate resistance to infection.

In addition to cells, the airways also have molecules, such as surfactants and hydrolases, that have been proposed as potential modulators of *M. tuberculosis* infection. Human surfactant proteins A and D bind to *M. tuberculosis* (62). Surfactant protein A upregulates expression of the mannose receptor on human macrophages (63), an important receptor for binding to *M. tuberculosis*, and modulates the inflammatory response of macrophages (64). Human surfactant protein D directly binds to *M. tuberculosis* and reduces the uptake by macrophages (65). However, mice deficient in both surfactant proteins A and D were not impaired in control of low-dose aerosol infection with *M. tuberculosis* (66). Antimicrobial peptides, such as cathelicidin (LL-37), are also present in airways (67) and have been shown to increase the proinflammatory functions of macrophages and the killing of intracellular *M. tuberculosis* (68). Intratracheal administration with synthetic peptides mimicking LL-37 in mice reduced *M. tuberculosis* bacterial burdens (69). There is substantial evidence that vitamin D is important in resistance to tuberculosis (70–73), and this appears to be in part due to induction of LL-37 (74, 75).

Antibodies are an obvious acquired immune response that might modulate the course of infection in airways. As part of the acquired immune response, pathogen-specific antibodies cannot be predicted to prevent initial infection in previously unexposed hosts, though it is possible that they could serve this function in the case of repeat exposure or vaccination. More importantly, although *M. tuberculosis* infection induces strong antibody responses, there is only scant experimental evidence that antibodies can prevent the initial establishment of infection. Passive transfer of antibodies specific to some cell wall antigens has been reported to confer protection against disease in a mouse model, but the effect was inconsistent. However, there are clear data that antibodies can change the interaction of the bacterium with macrophages in a variety of ways (76, 77); bacterial opsonization alters vesicular trafficking and macrophage signaling, and interactions of antibodies with activatory or inhibitory Fc receptors on macrophages can modulate macrophage function (76). Beyond their classical functions, antibodies have the capacity to mark the infected macrophage as aberrant and recruit the responses of other innate immune cells, thus making them potential modulators of the local immune response. However, whether antibodies are present in the airways in sufficient quantities to modulate initial infection remains unclear and is a source of substantial investigation.

In summary, there are a variety of cells, cytokines, and molecules present in airways that can modulate the initial response of the host to *M. tuberculosis* infection. These factors may prevent infection completely, limit initial establishment of granulomas, modulate the local environment of newly emerging granulomas, or increase the induction of T cell responses against *M. tuberculosis*. Changes in these factors could increase susceptibility to initial infection as well. Further studies will be necessary to more fully understand the relative contributions of these factors to modulation of initial infection.

### Lessons from clinical isolates of *M. tuberculosis.*

It is highly likely that bacterial factors drive differences in granuloma fate, as do host factors. Using barcoded bacteria to track origins of the bacterial populations in individual lesions, we have shown that most pulmonary granulomas arise from one progenitor bacterium (16), and genetic polymorphisms arise and become fixed in the bacterial populations of isolated granulomas (78). These data are consistent with historical data indicating that within a given individual, bacteria in one lesion can acquire drug resistance independently of the bacterial populations in other lesions (79). These data reinforce the concept that granulomas evolve relatively independently within the same host.

Many bacterial virulence factors have been identified through forward and reverse genetic approaches in experimental systems. However, it is not clear whether any of these might be modulated to alter interactions with the host in a lesion-specific fashion and thus contribute to the different lesional trajectories we and others have observed (4, 16, 80). This question has not yet been addressed through lesion-specific analyses, for example, transcriptional profiling of granuloma bacterial populations, which would be experimentally challenging given the relatively small number of bacteria in many lesions.

It is likely that the different virulence manifestations of clinical strains will shed some light on the bacterial pathways that flexibly alter interactions with the host. Six distinct lineages have now been defined based on sequence differences (81). There is mounting evidence that this genetic diversity generates clinically relevant phenotypic variation and impacts infection outcome. Strains have been shown to differ in terms of their mortality, pathological manifestations, and immune responses in mice and in human macrophages (82–85). Despite the mounting evidence that the genetic diversity of *M. tuberculosis* has clinical consequences, few concrete links between genotype and phenotype have been identified. The best-studied association has been between the presence of phenolic glycolipid biosynthesis and the hypervirulent phenotype and immunosuppressive properties associated with lineage 2 strains (86). In *M. marinum* infection of zebrafish, phenolic glycolipid promotes the recruitment of permissive macrophages to the site of infection and is required to establish robust infection (87). In modulating initial macrophage recruitment (85) as well as macrophage death pathways (discussed above), *M. tuberculosis* likely influences multiple early host interactions to affect inflammatory programs, granuloma fate, dissemination, and ultimately infection outcome.

It remains unclear whether bacterial expression of virulence lipids varies in a lesion-dependent fashion. It is interesting, however, that production of another virulence lipid, cell wall phthiocerol dimycocerosate (PDIM), is one mechanism to resolve propionyl-CoA toxicity during growth on fatty acids (88, 89). PDIM biosynthesis is required for bacterial survival in both mice and macrophages (90, 91), and PDIM has been proposed, based on work in *M. marinum*, to directly cloak Toll-like receptor 2 ligands (87). Thus, it is possible that the regulatory effects of central carbon metabolism, by which more or less PDIM may be produced depending on carbon source availability, provides an energy-efficient mechanism to link host environment with bacterial virulence and reinforce the trajectory of any given lesion after it is established by very early host events.

## CONCLUSIONS

Establishment and progression of *M. tuberculosis* remains somewhat of a mystery in humans. However, a deeper understanding of the early events in tuberculosis is essential to identifying new and effective strategies of preventing active TB. The best vaccine would prevent establishment of the infection, or at the very least prevent early dissemination of individual granulomas. Understanding the early airway and lung responses to this infection is crucial, as this is where control must occur. There are a variety of host cell types and molecules, as well as bacterial factors, which interact in early infection, as we have described here. Building on this knowledge will move the field of vaccines against tuberculosis forward. Without a clear understanding of the early processes that vaccines must prevent or limit and the host responses that can be harnessed for protection, we cannot expect a vaccine to succeed against this complex and evolved pathogen.
